# Relationships among the A Genomes of *Triticum* L. Species as Evidenced by SSR Markers, in Iran

**DOI:** 10.3390/ijms11114309

**Published:** 2010-11-02

**Authors:** Mohammad Hosein Ehtemam, Mohammad Reza Rahiminejad, Hojjatollah Saeidi, Badraldin Ebrahim Sayed Tabatabaei, Simon G. Krattinger, Beat Keller

**Affiliations:** 1 Department of Biology, University of Isfahan, Isfahan, 81746-73441, Iran; E-Mails: hehtemam@cc.iut.ac.ir (M.H.E.); ho.saeidi@sci.ui.ac.ir (H.S.); 2 Department of Agriculture, Isfahan University of Technology, Isfahan, 84156-83111, Iran; E-Mail: sayedt@cc.iut.ac.ir (B.E.S.T.); 3 Institute of Plant Biology, University of Zurich, Switzerland; E-Mails: skratt@botinst.uzh.ch (S.G.K.); bkeller@botinst.uzh.ch (B.K.)

**Keywords:** *Triticum*, SSRs, Iran, wheat, genetic analysis

## Abstract

The relationships among 55 wheat accessions (47 accessions collected from Iran and eight accessions provided by the Institute of Plant Biology of the University of Zurich, Switzerland) belonging to eight species carrying A genome (*Triticum monococcum* L., *T. boeoticum* Boiss., *T. urartu* Tumanian ex Gandilyan, *T. durum* Desf., *T. turgidum* L., *T. dicoccum* Schrank ex Schübler, *T. dicoccoides* (Körn. ex Asch. & Graebner) Schweinf. and *T. aestivum* L.) were evaluated using 31 A genome specific microsatellite markers. A high level of polymorphism was observed among the accessions studied (PIC = 0.77). The highest gene diversity was revealed among *T. durum* genotypes, while the lowest genetic variation was found in *T. dicoccoides* accessions. The analysis of molecular variance (AMOVA) showed a significant genetic variance (75.56%) among these accessions, representing a high intra-specific genetic diversity within *Triticum* taxa in Iran. However, such a variance was not observed among their ploidy levels. Based on the genetic similarity analysis, the accessions collected from Iran were divided into two main groups: diploids and polyploids. The genetic similarity among the diploid and polyploid species was 0.85 and 0.89 respectively. There were no significant differences in A genome diversity from different geographic regions. Based on the genetic diversity analyses, we consider there is value in a greater sampling of each species in Iran to discover useful genes for breeding purposes.

## 1. Introduction

The genus *Triticum* L. is one of the most important genera in the tribe Triticeae and has been the focus of many biosystematic studies. Four basic genomes, A, B, D and G are involved in the genomic constitution of all *Triticum* species [1,2]. The ancestral diploid species of A, B and D genome have diverged from a common ancestor about three million years ago [3]. From these ancestral diploids, two species hybridized somewhere along the Fertile Crescent to form the first tetraploid *Triticum* species [4]. The processes of polyploidization and genomic differentiation finally resulted in the present day genus *Triticum* with a ploidy series of di-, tetra- and hexaploid species, all based on x = 7 [5]. The A and D genomes which are less differentiated from those of the parental diploids, are considered as pivotal genomes [6,7]. Many reports indicated that the A genome has suffered different changes in *T. urarto* Thum. ex Gandil. (A^u^A^u^) and *T. boeoticum* Boiss. (A^b^A^b^) [2,8].

Since wheat cultivation commenced, the breeding and selection of particular genotypes have resulted in enormous loss of alleles and limited the genetic diversity of modern wheat cultivars [9,10]. Therefore, the remaining variability in the cultivated wheat gene pool is insufficient to address current and future breeding efforts [11]. For that reason, there is an essential and urgent need to explore the genetic potential among natural populations of wheat species and their closely related taxa. Germplasm accessions distinct from modern wheat cultivars are predicted to contain potentially useful alleles to broaden the genetic base of wheat [12].

Since the bread wheat (*T. aestivum*) most probably originated from the south eastern or south western Caspian Sea in Iran [13–15], the wild species and populations growing in Iran, as one of the putative centers of origin of cultivated wheat, can be valuable from this point of view. This opinion is strengthened by the fact that the chromosomes of A genome carry important genes such as adult plant resistance genes [16], milling yield genes [17], flour color genes[18], white salted noodle quality genes [19], supernumerary spikelet (SS) genes [20], sprouting resistance genes [21], chlorophyll synthesis genes [22], total florets per spike genes [23], cold tolerance genes [24], size of stomata genes [25], forest resistance genes [26,27] and yield traits such as tiller number, heading date and plant height genes [28]. Many workers have studied the *Triticum* species from different points of view: morphology [29,30], isozymes [14,31,32] restriction fragment length polymorphismes (RFLPs) [33–35], and microsatellites [36–39]. A high level of polymorphism in RFLPs and microsatellites among *Triticum* species accessions has been detected [37,40–43].

Microsatellites or simple sequence repeats (SSRs) have become the markers of choice among a variety of different molecular markers in order to evaluate genetic diversity and phylogenetic relationships [44,45]. It has been demonstrated that microsatellites are highly informative markers in many plant species [40,41,46–61] and it is believed that microsatellites show a much higher level of polymorphism in hexaploid wheat than any other marker systems.

More than a thousand wheat mapped microsatellite markers are available that are useful tools for genetic analyses. Genomic SSRs have been used in wheat for a variety of purposes including genomic mapping [33,40,62,63], gene tagging [39,64–66] and genetic diversity [41,67,68] analyses.

This study was aimed to use SSR markers to estimate the level of A genome polymorphism and to identify the relationships among the species carrying A genome of the genus *Triticum* native to Iran.

## 2. Results and Discussion

All 31 A genome specific SSR primers yielded 410 bands (alleles) from genomic DNA of all 55 accessions of eight A genome containing *Triticum* species from which 316 (0.77) were polymorphic (Table 1).

The number of alleles per microsatellite ranged from 5 (Xgwm512 and Xgwm179) to 22 (Xgwm666) with an average of 12.8 alleles per locus (Table 1). Major allele frequency ranged from 0.13 to 0.46 averaging 0.29 (Table 1). The mean value for polymorphism information content (PIC) for all microsatellites was 0.77. The microsatellite Xgwm427 with 20 alleles had the highest (0.92) and the microsatellite Xgwm136 with 6 alleles had the lowest (0.63) PIC value (Table 1).

### 2.1. Genetic Similarity Analysis

**Figure 1.**
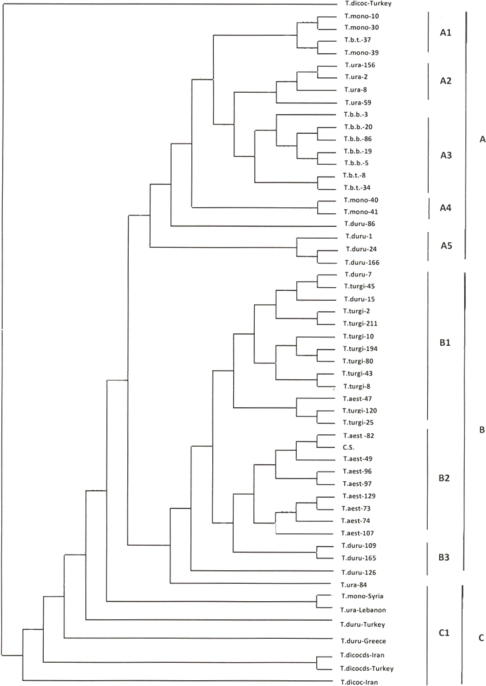
A genetic similarity based dendrogram showing relationships among *Triticum* accessions using 31 microsatellite markers. The main groups are denoted on the right side as A, B and C and the sub-groups as A1, A2, A3, A4, A5, B1, B2, B3 and C1. (T.mono = *Triticum monococcum*, T.b.t. = *T. boeoticum* subsp. *taodar*, T.b.b. = *T. boeoticum* subsp. *boeoticum*, T.ura = *T. urartu*, T.duru = *T. durum*, T.turgi. = *T. turgidum*, T.dicoc = *T. dicoccum*, T.dicocds = *T. dicoccoides*, T.aest = *T. aestivum*, and C.S. = Chinese spring).

The results distinguished all the 55 accessions (Figure 1), from which 46 were divided into two major groups designated as A and B in Figure 1 with 100% bootstrap support (data not shown).

These two groups, with several subgroups, were heterogeneous. The accessions of diploid species were grouped with considerable genetic similarities (except T.ura-84). Four accessions of tetraploid cultivated wheat *T. durum* were grouped with diploid accessions (group A, Figure 1). The group B included 14 tetraploid, 11 hexaploid and one diploid accession. The remaining eight accessions (provided by the Institute of Plant Biology, University of Zurich, Switzerland) were not grouped with the above main groups, and were clearly separated from the Iranian ones (group C, Figure 1).

At the species level (Table 2), the highest genetic similarity (0.89) was found between *T. aestivum* and *T. durum*; although *T. aestivum* and *T. turgidum* with a genetic similarity of 0.86 appeared relatively close too. The two species *T. dicoccum* and *T. dicoccoides* with 0.64 and 0.67 genetic similarity respectively, were grouped well away from the other species, indicating that the A genome in tetraploids was distant from the genome in the diploid and polyploid species.

In the UPGMA dendrogram (Figure 1), the eight *Triticum* species studied were divided into three groups: (1) three diploids (*T. monococcum*, *T. boeoticum*, *T. urartu*), (2) three cultivated wheats (*T. aetivum*, *T. durum* and *T. turgidum*), and (3) two tetraploids (*T. dicoccum* and *T. dicoccoides*).

### 2.2. Analysis of Molecular Variance (AMOVA)

The main portion of genetic variance (75.56%) was attributed to the variation among populations within species. A significant genetic variation (17.44%) was calculated between different species. There was no significant difference between A genome of species with different ploidy levels (Table 3).

**Figure 2.**
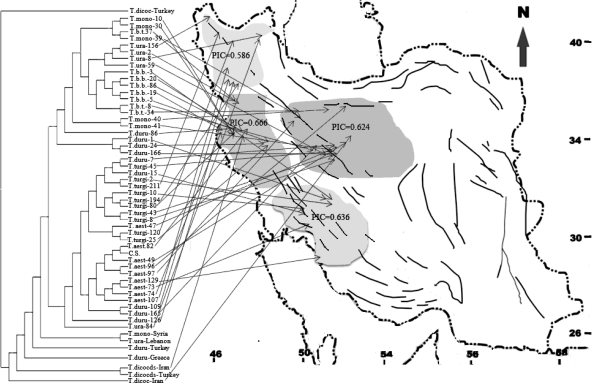
An integrated illustration of a dendrogram showing relationships among the accessions carrying A genome and the map of their geographic origin. Average PICs of all microsatellites are shown as average in each region.

The accessions were mainly collected from Iran as it is considered to be a part of centre of origin of cultivated wheat [15]. The germplasms presented in the centre of origin of a taxon are considered to be more diverse than those growing at the margins of its geographic distribution. The higher genetic diversity observed in this study concur with previous reports [37,41,42,69], indicating Iran as a likely part of the centre of diversity of this genus. The differences can also be attributed to the number of accessions studied, their genetic background, and the number of markers used. A total of 410 polymorphic bands (alleles) detected in this study seem to be enough to assess genetic variation among accessions. Zhang *et al.* (2002) [70] discussed that the presence of 350–400 alleles is enough for objective assessment of genetic relationship between wheat accessions. From the geographic point of view, the A genome SSR differentiations were not correlated with geographic distribution (Figure 2); however, some groupings related to both taxa and geographic origin (e.g., of *T. durum* accessions collected from NW and *T. boeoticum* subsp. *boeoticum* accessions collected from the West) were evident. The data were able to group geographically closely related collections (Figure 2). There were no significant differences between diversity measures calculated for different regions.

The recognition of the tetraploid species *T. durum* as a subspecies of *T. turgidum* by Kihara (1994) [1] and Mc Fadden and Sears (1966) [71] has been followed up by some other botanists. In spite of high genetic similarity between the two taxa showed in this study, they were enough apart to be considered as a distinct species; this is supported by morphological studies [72]. Analyses at the accessional level (Figure 1) indicated higher genetic distances among the accessions of these two species. The origin of the A genome encountered in the hexaploid wheat has always been under discussion, and its two closely related species, *i.e.*, *T. durum* and *T. turgidum,* have been generally known as the putative A genome donors to *T. aestivum* [71,73]. When one considers the very close genetic similarities between the above tetra- and the latter hexaploid *Triticum* species provided by this study (0.89 and 0.86 respectively, see Table 2), this notion is strengthened.

**Figure 3.**
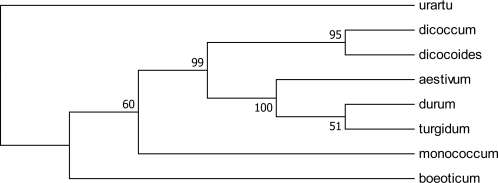
A bootstrap dendrogram based on genetic distances, constructed using UPGMA method, showing relationships between A genomes of 8 *Triticum* species.

Considering the topology of the UPGMA dendrogram (Figure 3), the tetraploid species were divided into two groups: (1) *T. turgidum* and *T. durum*, and (2) *T. dicoccoides* and *T. dicoccum*. This can be interpreted either as different post hybridization A genome modifications among the tetraploid species or involving two different origins as the A genome donor to them or preferential gene flow occurring between pair species within each group.

The SSR analysis showed a close relationship between the diploid *Triticum* species. Based on the calculated genetic similarities (Table 2), the A genomes occurring among the polyploids appeared to be more similar to that of the diploid species *T. monococcum* than the other diploids. This observation is partly in accordance with Johnson (1975) [74] and Tsunewaki (1999) [75] who pointed out that the A genome donor to *T. aestivum*, *T. turgidum* and *T. durum*, is *T. monococcum*, and to *T. dicoccoides* and *T. dicoccum* is *T. urartu*.

## 3. Experimental Section

A total of 47 accessions particularly collected for this study and eight accessions provided by the Institute of Plant Biology, University of Zurich (Table 4) were examined.

The materials were taxonomically identified based on Rahiminejad and Kharazyan [72]. DNA was isolated from fresh leaves of twenty individuals of each accession using CTAB DNA extraction method [76]. In order to assess genetic relationships and diversity of the species carrying A genome in the genus *Triticum*, 31 A genome specific SSR primers [36] were used. Markers name and other details regarding the SSR markers are presented in Table 1.

Polymerase chain reactions (PCRs) were performed based on Jakson and Matthews (2000) [77]. Briefly, the PCR was carried out in 10 μL containing 2.5 μL of the 13 ng/μL genomic DNA sample, 1 μL of 10 × reaction buffer, 0.3 μL of 30 mM MgCl_2_, 1 U *Taq* polymerase, 0.5 μL of 2.5 mM dNTPs and 0.3 pmol each of IRD 800 dye and IRD 700 dye labeled arbitrary primers. The amplification program consisted of the following cycles: 95 °C for 4 min, 95 °C for 30 sec, 50 °C to 60 °C (depending on the primer set) for 30 sec, 72 °C for 1.5 min and a final extension at 72 °C for 10 min. After 4 min at 95 °C, 35 cycles were performed for 30 sec at 95 °C, 30 sec at annealing temperature (50–60 °C, see Table 1), 1.5 min at 72 °C, followed by a final extension step of 10 min at 72 °C. Upon completing the PCR cycles, 0.8 μL of PCR products of each sample was loaded onto a 6% polyacrylamide sequencing gel in a Li-COR Global DNA Sequencer, and electrophoresed at 2000 V for 1.5 h.

### Statistical Analyses

In order to perform a simple phenetic analysis, the presence (1) and absence (0) of each band with particular mobility was scored. Genetic similarities were calculated using Powermarker [78] and NTSYS-PC softwares from the data tables. A genetic similarity based dendrogram (based on Jaccard’s similarity coefficient) [56] was constructed to show relationships between populations and species using the UPGMA clustering method implemented in NTSYS-PC [79].

For the genetic diversity analysis, allele number per locus, major allele frequencies, polymorphism information content ( $PIC=1-\sum_{i=1}^{n}p_{i}^{2}-\sum_{i=1}^{n-1}\sum_{j=i+1}^{n}2\overset{2}{\underset{i}{p}}\overset{2}{\underset{j}{p}}$, where *p_i_* and *p_j_* are the frequencies of the *i*th and *j*th alleles of a given marker, respectively [80]) and gene diversity (*D_i_*) were calculated as genetic parameters of polymorphism for each marker in all accessions, and for all markers in the accessions collected from the same geographical regions (see Figure 2). For all the parameters, the overall estimates were calculated as the averages across all loci. The analysis of molecular variance (AMOVA; implemented in ARLEQUIN software [81]) was carried out to estimate the variance components of fingerprinting patterns, and to partition the variation among ploidy levels (groups), among the species with a same ploidy level (within groups) and among the accessions within the species.

## 4. Conclusion

Based on the results of this study, and those previously reported [82,83], it can be concluded that a hypothetical process of hybridization, polyploidization and genomic differentiation can result in the A genome of bread wheat as below:

T. monococcum  → T. durum:T. turgidum  → T. aestivum

Huang *et al.* (2002) [29], and Wicker *et al.* (2003) [84] suggested that the genome of *T. urartu* (A^u^) diverged about 0.5–3 Mya from the genome of *T. monococcum* (A^m^). Therefore, it can be proposed that *T. monococcum* is the parental A genome species in the genus *Triticum* and the A genome of di-, tetra- and hexaploid species carrying A genome are directly or indirectly originated from this species. The populations of *T. monococcum* grow in restricted areas and the data (not shown) indicated low seed germination ability (about 10%) of this species. Therefore, this can be interpreted that *T. monococcum* is facing the risk of extinction in this area and we suggest that carrying out a wider sampling with more depth across its geographic range of distribution would be vital for gene pool conservation proposes.

As Figure 1 shows the materials provided by the Institute of Plant Biology, University of Zurich are clearly separated from the Iranian accessions. These originated from Turkey, Syria, Lebanon, Greece and Iran. In dendrogram, these accessions were mainly divided according to their country of origin. The *T. dicoccoides* accessions originating from Iran and Turkey were grouped together; however, because of less materials being studied no interpretation can be made about their genetic relationship. Since there were no materials belonging to *T. dicoccum* and *T. dicoccoides* among the 47 accessions specifically collected from Iran for this study, no comparison was made with their Iranian genepool (Table 4).

AMOVA analyses showed that the main portion of diversity is attributed to the variation among the accessions within species, suggesting high genetic diversity within the *Triticum* species in Iran. We would expect that a great sampling of each species in Iran and analysis of genetic diversity would be worthwhile to reveal the genetic structure of their gene pools and to discover new useful alleles for breeding proposes.

## Figures and Tables

**Table 1.** Amplification of the homologous microsatellites in 55 accessions of the genus *Triticum* using 31 primer sets originally designed for the microsatellites of A genome (for the primer sequence see Röder *et al.* 1998 [36]).

**Marker**	**Chr. Loc.**	**Ann. Temp.**	**Allele Fr.**	**Allele No**	**HE**	**HO**	**PIC**
gwm-601	4A	60	0.37	15	0.7	0.85	0.66
gwm-135	1A	60	0.28	14	0.84	0.66	0.83
gwm-71	2A	60	0.22	18	0.86	0.86	0.85
gwm-666	1A, 3A, 5A, 7A	60	0.22	22	0.86	0.98	0.84
gwm-311	2A, 2B, 6B	60	0.23	12	0.86	0.27	0.85
gwm-359	2A	55	0.23	14	0.86	0.75	0.85
gwm-512	2A	60	0.29	5	0.75	0.12	0.70
gwm-372	2A	60	0.23	14	0.88	0.24	0.87
gwm-391	3A	55	0.22	18	0.79	0.81	0.77
gwm-757	3A	60	0.27	14	0.84	0.74	0.83
gwm-155	3A	60	0.34	8	0.77	0	0.74
gwm-291	5A	60	0.41	16	0.77	0.59	0.74
gwm-494	6A, 4A, 3A, 1B	60	0.34	12	0.77	0.87	0.74
gwm-427	6A	50	0.13	20	0.92	0.24	0.92
gwm-635	7A, 7B, 7D	60	0.20	11	0.86	0.63	0.85
gwm-332	7A	60	0.23	12	0.85	0.67	0.84
gwm-296	2A, 2D, 7D	55	0.14	18	0.78	0.49	0.76
gwm-471	7A, 7B	60	0.26	12	0.85	0.39	0.84
gwm-260	7A	55	0.21	13	0.87	0.83	0.86
gwm-459	6A	55	0.46	9	0.73	0.25	0.71
gwm-179	5A	55	0.31	5	0.77	0.62	0.74
gwm-382	2A, 2B, 2D	60	0.27	15	0.86	0.26	0.85
gwm-205	5A, 5D	60	0.23	19	0.9	0.8	0.89
gwm-136	1A	60	0.41	6	0.68	0.5	0.63
wmc-104	1A, 6B	55	0.44	10	0.74	0.22	0.72
barc-56	5A	55	0.32	15	0.78	0.5	0.75
barc-151	5A, 7A	55	0.19	13	0.88	0.14	0.87
cfa-2086	2A	60	0.19	17	0.86	0.46	0.85
cfa-2028	7A	55	0.33	9	0.75	0.72	0.72
cfa-2262	3A	55	0.22	13	0.77	0.2	0.74
cfa-2263	2A	60	0.17	11	0.88	0.25	0.87

**Mean**			0.29	12.8	0.79	0.49	0.77

**Sum**				410			

**Table 2.** The analysis of genetic similarity between A genomes of diploid and diploid, diploid and tetraploid, diploid and hexaploid, tetraploid and tetraploid, and tetraploid and hexaploid pair species of 55 accessions belonging to eight *Triticum* L. species as revealed by SSR markers.

**Groups**	**Species**	**Genetic Similarity**
**diplo & diplo**	*T. monococcum & T.boeoticum*	0.89
	*T. monococcum & T. urartu*	0.90
	*T.boeoticum & T. urartu*	0.90
**tetra & tetra**	*T. durum & T. turgidum*	0.86
	*T. durum & T. dicoccum*	0.79
	*T. durum & T. dicoccoides*	0.78
	*T. turgidum & T. dicoccum*	0.79
	*T. turgidum & T. dicoccoides*	0.78
	*T. dicoccum & T. dicoccoides*	0.70
**diplo & tetra**	*T. monococcum & T. durum*	0.85
	*T. monococcum & T. turgidum*	0.66
	*T. monococcum & T. dicoccum*	0.74
	*T.monococcum&T.dicoccoides*	0.74
	*T. boeoticum & T. durum*	0.82
	*T. boeoticum & T. turgidum*	0.65
	*T. boeoticum & T. dicoccum*	0.66
	*T. boeoticum & T. dicoccoides*	0.69
	*T. urartu & T. durum*	0.84
	*T. urartu & T. turgidum*	0.64
	*T. urartu & T. dicoccum*	0.75
	*T. urartu & T. dicoccoides*	0.76
**diplo & hexa**	*T. monococcum & T. aestivum*	0.77
	*T. boeoticum & T. aestivum*	0.72
	*T. urartu & T. aestivum*	0.72
**tetra & hexa**	*T. durum & T. aestivum*	0.89
	*T. turgidum & T. aestivum*	0.86
	*T. dicoccum & T. aestivum*	0.64
	*T. dicoccoides & T. aestivum*	0.67

**Table 3.** The analysis of molecular variance (AMOVA) of 55 accessions of eight A genome containing species of the genus *Triticum* calculated at ploidy level (groups), species within each ploidy level (within groups) and accessions of each species (within species).

**Source of variation**	**d.f**	**Sum of squares**	**Mean of squares**	**Percentage of variation**	**Variance components**	**P-value**
**Among Ploidy levels (groups)**	2	235.865	*117.932*	7.00	2.30879	<0.001
**Among species Within groups**	5	286.738	57.347	17.44	5.75630	<0.001
**Among accessions**	47	1172.161	24.939	75.56	24.93959	0.10948 ± 0.00939

**Total**	54	1694.764	200.218	100	33.00468	

**Table 4.** The species name, collection label, genome combination and the origin of accessions used in this study.

**Species**	**Collection label**	**Genome**	**Locality and altitude (m)**
*T. monococcum*	T. mono-30	A	Kermanshah, Gardaneh Reno (1480)
	T. mono -10	A	Kordestan, 3 km to Saghez (1620)
	T. mono -41	A	Isfahan, Semirom to yasooj (2100)
	T. mono -39	A	Arak to Malayer (2020)
	T. mono -40	A	Tehran, Taleghan valley (1850)
	T. mono Syria	A	Provided by Institute of Plant Biology of the University of Zurich
*T.boeoticum* subsp*. thaodar*	T.b.t.-37	A	Kordestan, 5 km after Jenan to Saghez (1770)
	T.b.t.-8	A	Chaharmahal Bakhtiari, Shahr-e-Kord, Shapoorabad to Jooneghan (2090)
	T.b.t.-34	A	Arak 15 km to Malayer (1840)
*T.boeoticum* subsp. *boeoticum*	T.b.b.-19	A	Ilam to Kermanshah, Gardaneh Reno (1370)
	T.b.b.-5	A	Lorestan, 35 km to Khoramabad from Malavi (1100)
	T.b.b.-20	A	Kermanshah 10 km to Harsin (1330)
	T.b.b.-86	A	Kermanshah to Kamyaran (1340)
	T.b.b.-3	A	Kohkiloye & Boyerahmad,Yasooj, Amirabad (1650)
*T.urartu*	T.ura-156	A	West Azarbaijan, Makoo (1580)
	T.ura-84	A	Ardabil (1320)
	T.ura-2	A	Kordestan, 10 km Saghez from Asadabad (1440)
	T.ura-8	A	Aradbil, 10 km to Kaghazkanan (1349)
	T.ura-59	A	Chaharmahal Bakhtiari, between Gandoman and Lordegan (2080)
	T.ura-Lebanon	A	Provided by Institute of Plant Biology of the University of Zurich
*T.durum*	T.duru-86	AB	Kermanshah, Kamyaran (1440)
	T.duru-24	AB	Lorestan, Malavi toward Khoram Abad (1200)
	T.duru-166	AB	Chahar mahal Bakhtiari, DoAb Samsami (2000)
	T.duru-1	AB	Kohkiloye & Boyerahmad (990)
	T.duru-165	AB	Chahar mahal Bakhtiari, near Chaghakhor lake (2190)
	T.duru-109	AB	West Azarbaijan, Sardasht to Baneh (1050)
	T.duru-15	AB	Khoosestan, Haftgel to Masjed Soleiman (550)
	T.duru-126	AB	Kordestan, Alamoot 6 Km (1660)
	T.duru-7	AB	Chahar mahal Bakhtiari, Borojen to Izeh (2190)
	T.duru-Turky	AB	Provided by Institute of Plant Biology of the University of Zurich
	T.duru-Greece	AB	Provided by Institute of Plant Biology of the University of Zurich
*T. turgidum*	T.turgi-211	AB	West Azarbaijan, Khoi (1110)
	T.turgi-45	AB	Chahar mahal Bakhtiari, Bazoft (2190)
	T.turgi-2	AB	Kohkiloye & Boyerahmad, Yasooj (2880)
	T.turgi-43	AB	Chahar mahal Bakhtiari, Bazoft, Morez valley (2000)
	T.turgi-8	AB	Chahar mahal Bakhtiari, Borojen to Izeh (2190)
	T.turgi-10	AB	Khoosestan, Izeh (900)
	T.turgi-194	AB	Kordestan, between Sanandaj and Saghez (1595)
	T.turgi-80	AB	Kermanshah, Mahi Dasht (1290)
	T.turgi-25	AB	Lorestan, Malavi toward Khoram Abad (1200)
	T.turgi-120	AB	East Azarbaijan, Ahar (1320)
*T. dicoccum*	T.dicoc-Turkey	AB	Provided by Institute of Plant Biology of the University of Zurich
	T.dicoc-Iran (Isfahan)	AB	Provided by Institute of Plant Biology of the University of Zurich
*T. dicoccoides*	T.dicocds-Iran (Kermanshah)	AB	Provided by Institute of Plant Biology of the University of Zurich
	T.dicocds-Turkey	AB	Provided by Institute of Plant Biology of the University of Zurich
*T. aestivum*	T.aest-47	ABD	Chahar mahal Bakhtiari (2000)
	T.aest-74	ABD	Ilam, Do Rahe (1410)
	T.aest-129	ABD	Booshehr, Bandargah to Deilam (17)
	T.aest-73	ABD	Khoosestan, Karkheh (13)
	T.aest-97	ABD	Mlayer toward Arak, 50Km (2010)
	T.aest-96	ABD	Tehran, Firooz kouh1 (700)
	T.aest-107	ABD	West Azarbaijan, Boukan to Mahabad (1290)
	T.aest-49	ABD	Isfahan, Daran (2190)
	T.aest-82	ABD	Kermanshah, Mahi Dasht (1290)
Chinese spring	C.S.	ABD	Provided by Institute of Plant Biology of the University of Zurich
